# Oral Alitretinoin for Patients with Refractory Prurigo

**DOI:** 10.3390/medicina56110599

**Published:** 2020-11-09

**Authors:** Bo Young Chung, Ji Young Um, Seok Young Kang, Min Je Jung, Jin Cheol Kim, In-Suk Kwak, Chun Wook Park, Hye One Kim

**Affiliations:** 1 Department of Dermatology, Kangnam Sacred Heart Hospital, Hallym University college of Medicine, 1, Singil-ro, Yeoungdeungpo-gu, Seoul 07441, Korea; victoryby@naver.com (B.Y.C.); ujy0402@hanmail.net (J.Y.U.); tjdjrdud@naver.com (S.Y.K.); luckyminja77@naver.com (M.J.J.); aiekfne@naver.com (J.C.K.); 2 Department of Anesthesiology and Pain Medicine, Burn Center, Hangang Sacred Heart Hospital, Hallym University College of Medicine, 94-200 Yeoungdeungpo-dong, Yeoungdeungpo-gu, Seoul 07247, Korea; 031132@hallym.or.kr

**Keywords:** prurigo, alitretinoin, pruritus, refractory

## Abstract

*Background*: prurigo is a chronic skin disorder associated with a history of chronic pruritus. The pathogenesis of prurigo is largely unknown and the treatment of prurigo is unsatisfactory and challenging. Conventional systemic treatments may be beneficial; however, their possible side effects and possible transient efficacy is still a problem. We aimed to present the clinical course and effect of treatment with alitretinoin on patients with prurigo nodularis initially treated with conventional treatments like oral antihistamine, cyclosporine, and phototherapy. *Methods*: all the patients had prurigo nodularis refractory to conventional treatment. Their medical records included demographic features, past medical history, duration of disease, and treatment modalities; and the clinical courses of the patients were reviewed for this retrospective study. We evaluated patient pruritus and skin lesions for the duration. *Results*: we present reports involving 10 patients with refractory prurigo. All the patients in our cases were treated with oral alitretinoin after previous treatments and reported the improvement of skin lesions and pruritus within 2 weeks to 3 months. *Conclusions*: we suggest that oral alitretinoin may be an effective and well tolerated treatment option for patients with intractable prurigo. Further clinical studies are warranted to confirm the long-lasting efficacy and safety of alitretinoin for treating patients with prurigo.

## 1. Introduction

Prurigo is a chronic skin disorder associated with a history of chronic pruritus [1]. This is the group of skin diseases characterized by intensely pruritic papules and nodules with visible excoriations and ulcerations due to scratching [2]. It has many variations such as papular dermatitis, subacute prurigo, itchy red bumps, and acquired perforating dermatosis (Kyrle’s diseases). There are different clinical subtypes of chronic prurigo that can be identified according to the predominate lesion: papular prurigo, prurigo nodularis, plaque-type prurigo, umbilicated prurigo, and linear prurigo. Chronic and repetitive scratching, picking, or the rubbing of the nodules may result in permanent changes to the skin, including lichenification, hyperkeratosis, hyperpigmentation, and skin thickening. Unhealed, excoriated lesions are often scaly, crusted or scabbed. Many patients report a lack of wound healing even when medications relieve the itching and subsequent scratching. The pathogenesis of prurigo is largely unknown and the treatment of prurigo is unsatisfactory and challenging [2]. Conventional systemic treatments like oral antihistamine, cyclosporine, and phototherapy may be beneficial for patients who fail to respond to topical and intralesional corticosteroids; however, their possible side effects and possible transient efficacy, as well as the chronic and intractable characteristics of this disease, make us feel the need for alternative treatments [2]. There have been reports of several cases in which alitretinoin showed good clinical response in chronic inflammatory diseases such as lichen planus, and chronic hand eczema [2,3,4,5]. In addition, the intensity of itching significantly decreased after treatment with alitretinoin in several cases of patients with prurigo nodularis [2,6]. Here, we report a 10-case series of patients suffering from chronic itching with refractory prurigo, who were successfully treated with alitretinoin.

## 2. Materials and Methods

This study included Korean prurigo patients who attended the Department of Dermatology, Hallym University, Kangnam Sacred Heart Hospital, between March 2014 and December 2018. In this case series, we examined the clinical course and effect of treatment with alitretinoin on patients with prurigo nodularis. All the patients reviewed had prurigo nodularis refractory to conventional treatment. Their medical records included demographic features, past medical history, the duration of disease, and treatment modalities; and the clinical courses of the patients were reviewed for this retrospective study. We evaluated patient pruritus and skin lesions for the duration of their stays in our outpatient clinic. We called the patients and asked if the pruritic lesions and pruritus recurred after 3 months or more. Complete remission in this report is defined as improved pruritus and skin lesions after treatment and for which treatment was terminated without recurrence or additional outpatient visits. Partial remission is defined as improved pruritus and skin lesions after alitretinoin treatment, compared with those after conventional treatments.

## 3. Results

We present reports involving 10 patients with refractory prurigo, who suffered from skin lesions with chronic pruritus (Table 1).

Most of the patients were diagnosed based on histological evidence and given conventional treatments like oral antihistamine, cyclosporine, and phototherapy; however, the effect of treatment was insufficient and some patients could not continue treatment due to side effects. After their previous treatments, all the patients in our cases were treated with oral alitretinoin and reported an improvement of skin lesions and pruritus within 2 weeks to 3 months (Table 2).

### 3.1. Case 1

**Figure 1:**
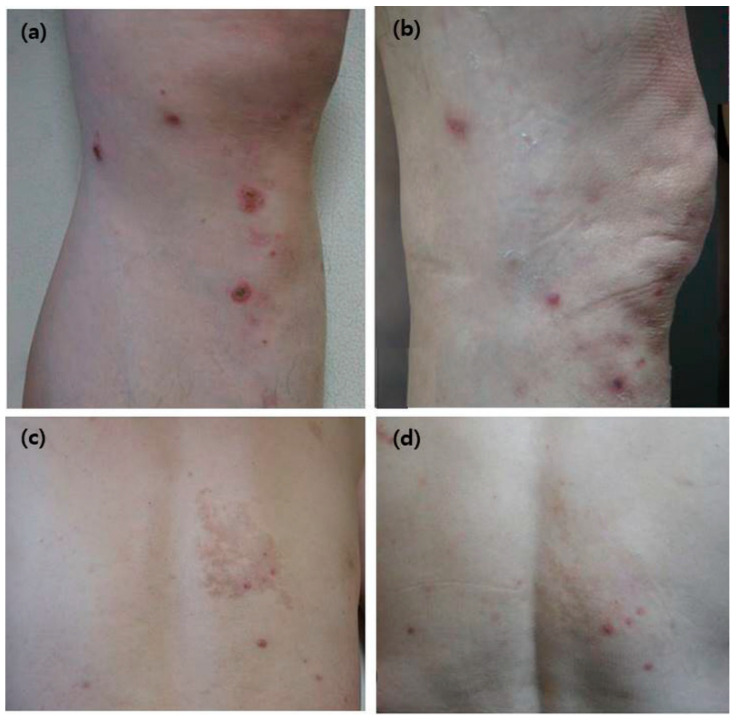
Representative clinical manifestation after treatment with alitretinoin in Case 1 (61 year-old man), a patient with prurigo nodularis: (**a**,**c**) multiple erythematous to brownish papules and nodules on the back and both legs (**b**,**d**).

**Figure 2:**
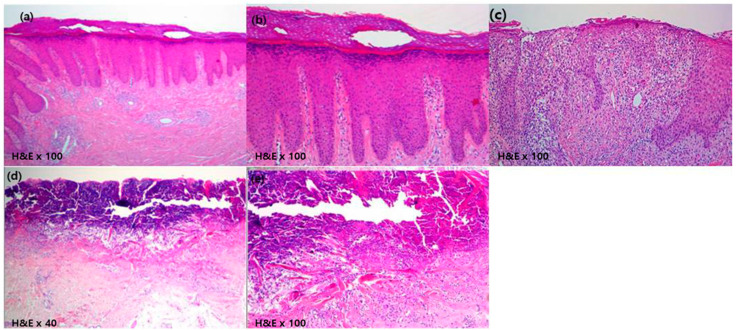
Pathology of skin lesions in patients with prurigo nodularis. (**a**,**b**) Microscopic examination shows hyperkeratotic epidermis with acanthosis and parakeratosis (Hematoxylin & Eosin (H&E), ×40). Elongated and irregular rete ridges are observed with dense dermal perivascular infiltration (H&E, ×100), Case 1 (61 year-old man). (**c**) Microscopic examination showed a pronounced granular layer and numerous lymphocytic infiltrations of the papillary dermis with mild spongiosis (H&E, ×100), Case 9 (56 year-old man). (**d**,**e**) Cup-shaped invagination of the epidermis is associated with a keratin plug containing inflammatory debris and collagen fibers (H&E, ×40 and ×100), Case 10 (41 year-old woman).

A 61 year-old man presented with several one month-old erythematous to brownish papules and nodules on the back and both legs (Figure 1a,c). He had diabetes mellitus. The initial clinical suspicion was prurigo nodularis and punch biopsy was done. Microscopic examination showed hyperkeratotic epidermis with acanthosis and parakeratosis. Elongated and irregular rete ridges were observed with dense dermal perivascular infiltration (Figure 2a,b). The final diagnosis was prurigo nodularis. Initially, he was treated with oral cyclosporine 100 mg/day for 4 weeks. However, relief of the symptoms was insufficient and oral alitretinoin was started at a dose of 30 mg/day. After 4 weeks of alitretinoin treatment, symptoms and skin lesions were improved, and with 8 weeks of treatment (Figure 1b,d), he stopped medical treatment and achieved complete remission without recurrence.

### 3.2. Case 2

A 65 year-old man had developed multiple, scattered, itchy excoriated erythematous papules on both arms starting 2 years prior. He had no underlying disease. The biopsied skin lesion showed hyperkeratotic epidermis with acanthosis. Elongated and irregular rete ridges were observed. The final diagnosis was prurigo. He was treated with oral cyclosporine (25 mg/day) and naltrexone (50 mg/day), and there was no improvement in the symptoms within 2 years. Therefore, the oral alitretinoin treatment (30 mg/day) was started. After 3 weeks of alitretinoin treatment, the patient achieved partial remission. The skin lesions and itching sensation were improved and the patient stopped medical treatment.

### 3.3. Case 3

A 75 year-old man presented with multiple scattered nodules and excoriated patches with crusts on the trunk, both arms, and both legs that were two weeks old. He had no underlying disease. The initial clinical diagnosis was prurigo nodularis and treatment was started. Despite treatment with oral cyclosporine (100 mg/day) for 3 years, his symptoms and skin lesions waxed and waned. After that, the treatment was changed to oral alitretinoin in a dose of 30 mg/day. Within 12 weeks of treatment, pruritus had improved and treatment with oral alitretinoin was stopped with partial remission.

### 3.4. Case 4

A 41 year-old woman presented with localized brownish papules and patches with crust and a scaly skin lesion. She had chronic kidney disease. Her skin lesion was diagnosed as prurigo nodularis. Because of her impaired kidney function, medication that might impair renal function was limited. Treatment with oral alitretinoin was selected in a dose of 30 mg/day. Within 4 weeks of treatment, the symptoms had improved compared to conventional treatment. She continued treatment with oral alitretinoin during which the symptoms waxed and waned.

### 3.5. Case 5

A 42 year-old man presented with multiple scaly erythematous itchy papules on the whole body that were 12 months old. He had hypertension and diabetes mellitus. The clinical diagnosis was prurigo nodularis and treatment was started. Despite taking oral antihistamine and cyclosporine (200 mg/day) for 4 weeks, the patient’s symptoms were not improved. Medication was changed to oral alitretinoin in a dose of 30 mg/day. Within 5 weeks of treatment, symptoms had improved. The treatment of oral alitretinoin was continued for 12 weeks until complete remission and was stopped afterward without recurrence.

### 3.6. Case 6

A 78 year-old man diagnosed with prurigo nodularis had itchy multiple erythematous to brownish papules on both flanks, chest, and back that were 6 months old. He had no underlying disease. Oral cyclosporine (50 mg/day) was taken for 1 year. His symptoms waxed and waned. In our study, oral alitretinoin (30 mg/day) was started. Within 2 weeks of treatment, the skin lesions and pruritus had improved. The patient was treated with oral alitretinoin for 14 weeks until complete remission and then the treatment was stopped without recurrence.

### 3.7. Case 7

A 54 year-old man presented with multiple, 1 month-old, itchy erythematous maculopapular patches on both legs, both arms, and back, which were diagnosed as prurigo nodularis. He had no underlying disease. Treatment with oral cyclosporine (50 mg/day) was started. However, his blood pressure exceeded the hypertension borderline after 8 weeks of treatment even though he had no underlying hypertension. Therefore, the treatment was changed to oral alitretinoin (30 mg/day) for 2 weeks. His itching sensation was reduced after the treatment without recurrence.

### 3.8. Case 8

A 54 year-old man had developed several scattered, itchy, excoriated erythematous papules on both the arms and back (5 years previously). He had no underlying disease. The clinical diagnosis was prurigo nodularis and treatment was started. Oral cyclosporine (200 mg/day) was taken for 1 year, but his symptoms waxed and waned. Treatment was changed to oral alitretinoin (30 mg/day). Within 16 weeks of treatment, the symptoms and skin lesions had improved more than with previous treatment. His treatment with oral alitretinoin continued for 3 years without progression or any side effects.

### 3.9. Case 9

A 56 year-old man presented with 2 year-old, itchy, erythematous to brownish papules with silver scales on both legs. He had hypertension and diabetes mellitus. The clinical diagnosis was prurigo nodularis and punch biopsy was done. Microscopic examination showed a pronounced granular layer and numerous lymphocytic infiltrations of the papillary dermis with mild spongiosis (Figure 2c). The final diagnosis was Kyrle’s disease with prurigo nodularis. He was treated with cyclosporin (100 mg/day) and narrowband ultraviolet-B(NB-UVB) twice/week for 12 weeks. However, there was no improvement of symptoms. Therefore, oral alitretinoin (30 mg/day) was started. After 16 weeks of treatment, skin lesions and pruritus were improved. His score on the visual analog scale for pruritus improved (from 6 to 3). His treatment with oral alitretinoin continued for 19 months and he successfully achieved complete remission without recurrence.

### 3.10. Case 10

A 41 year-old woman presented with multiple, 2 year-old, scattered erythematous to brownish papules with pruritus on both legs. She had diabetes mellitus. The initial clinical suspicion was reactive perforating collagenosis and punch biopsy was performed. Atrophic and ulcerative epidermis was observed. There was cup-shaped invagination of the epidermis associated with a keratin plug containing inflammatory debris and collagen fibers (Figure 2d,e). Reactive perforating collagenosis and prurigo nodularis were finally diagnosed. After the use of oral cyclosporine (100 mg/day) for 4 weeks without improvement, oral alitretinoin (30 mg/day) was started. Within 5 weeks of alitretinoin treatment, her pruritus and skin lesions improved. After that, she stopped medical treatment without progression or recurrence.

## 4. Discussion

The treatment of prurigo is unsatisfactory and challenging [2]. Conventional systemic treatment with methotrexate, cyclosporine, thalidomide, and gabapentin for patients who fail to respond to topical and intralesional corticosteroids and phototherapy may be beneficial. However, their use comes with possible side effects, (and with cyclosporine in particular) adverse effects such as nephrotoxicity and increase in blood pressure and possible transient efficacy. These effects, as well as the chronic and intractable characteristics of this disease, made us feel the need for alternative treatments [2].

Alitretinoin (9-cis retinoic acid) is a unique panagonist vitamin A derivative, which binds to both retinoic acid receptors (RAR) and retinoid X receptors (RXR) [7,8]. Thus, alitretinoin could act upon a wider spectrum of diseases and in different pathways compared to the old retinoids. It has anti-inflammatory and immunomodulating effects and has been demonstrated to regulate the production of cytokine and leukocyte activity [8]. These effects of alitretinoin are exerted by the suppression of chemokine receptor expression and the disturbing recruitment of inflammatory cells [9]. The histological examination of prurigo samples presented an accumulation of neuropeptides that upregulated the production of many proinflammatory cytokines (interleukin (IL)-1α, IL-1β, and IL-8) [10]. There was also a degranulation of a substantial number of mast cells with the subsequent release of histamine. This induces pruritus, which stimulates the proliferation of fibroblasts and the synthesis of collagen [10]. In vitro, alitretinoin inhibited the production of nitric oxide and proinflammatory cytokines such as tumor necrosis factor (TNF)-α, IL-1β, and IL-12p40. It also increases the production of IL-4 by human T cells [8,11]. Type 1 helper T cells (Th1), IL-4/IL-13, and Th2 cytokines interferone-γ are assumed to be factors inducing the spongiotic epidermal changes in prurigo nodularis in the course of disease progression. Accordingly, Th1 and Th2 cytokines have also been evaluated for roles in the pathogenesis of prurigo [7,8,11,12]. Furthermore, alitretinoin also markedly reduced the number of macrophages and activated dendritic cells, which are two major sources of TNF-α, and hence considerably reduce inflammation [13]. Other inflammatory chemokines of which the levels are diminished by alitretinoin include IL-4, IL-1β, and IL-12p40 [3,8]. Alitretinoin also inhibits the production of nitric oxide (NO), secondary to stimulation by other cytokines and lipopolysaccharides, and furthermore, brings about immunomodulation by downregulating the activity of leucocytes [14]. 

Recent research on alitretinoin showed effective clinical response for several chronic inflammatory diseases: chronic hand eczema, lichen planus, lichen simplex chronicus, atopic dermatitis, palmoplantar pustular psoriasis, pityriasis rubra pilaris, and Darier’s disease [15,16,17,18]. Regarding prurigo, in one case involving a 46 year-old Caucasian woman, it was reported that therapy with oral alitretinoin led to a substantial clinical improvement of skin manifestations with pruritus [2]. A significant decrease in the intensity of itch after treatment with alitretinoin has already been demonstrated in the literature [2]. The possible adverse events in patients taking alitretinoin include headache, dry skin, rash, alopecia, exfoliative dermatitis, hyperlipidemia, and teratogenic effects [6]. In the current study, we explained the side effects of drugs to the patients, and in the case of long-term use, periodic blood tests were performed every two months. All patients presented any specific side effects other than the most common side effect, initial headache and most were tolerable.

Here, we presented a 10-case series of refractory prurigo patients initially treated with conventional treatments like oral antihistamine, cyclosporine, and phototherapy, but for which the treatment effect was insufficient. In some cases, the patients could not continue treatment due to side effects. All the patients in our cases were treated with oral alitretinoin after previous treatment and reported the improvement of skin lesions and pruritus within 2 weeks to 3 months. We suggest that oral alitretinoin may be an effective and well tolerated treatment option for patients with intractable prurigo. The exact effects of alitretinoin in the pathway of pruritus and pathogenesis of prurigo are still unclear; so further analysis with the pharmacology of alitretinoin and the mechanism of effects in clinical diseases are needed to confirm our clinical observations.

## 5. Conclusions

We report that alitretinoin offers another option for the treatment of refractory prurigo. Further clinical studies are warranted to confirm the long-lasting efficacy and safety of alitretinoin for treating patients with prurigo.

## Figures and Tables

**Table 1:** Demographic and clinical features of the patients in the current study.

Patient No.	Sex/Age (yrs)	Duration	Diagnosis	Underlying Disease
Case 1	M/61	4 weeks	Prurigo nodularis	DM
Case 2	M/65	24 weeks	Prurigo nodularis	None
Case 3	M/75	2 weeks	Prurigo nodularis	None
Case 4	F/41	4 weeks	Prurigo nodularis	CKD
Case 5	M/42	48 weeks	Prurigo nodularis	HTN/DM
Case 6	M/78	72 weeks	Prurigo nodularis	None
Case 7	M/54	4 weeks	Prurigo nodularis	None
Case 8	M/54	60 weeks	Prurigo nodularis	None
Case 9	M/56	96 weeks	Kyrle’s disease, Prurigo nodularis	HTN/DM
Case 10	F/41	8 weeks	Reactive perforating collagenosis, Prurigo nodularis	DM

HTN: hypertension, DM: diabetes mellitus, CKD: chronic kidney disease.

**Table 2:** Treatment course of patients in current study.

Patient No.	Duration for Conventional Treatments	Time to Remission after Alitretinoin	Clinical Course
Case 1	4 weeks	8 weeks	Complete remission
Case 2	104 weeks	3 weeks	Partial remission
Case 3	156 weeks	208 weeks	Partial remission
Case 4	312 weeks	4 weeks	Partial remission
Case 5	4 weeks	12 weeks	Complete remission
Case 6	52 weeks	14 weeks	Complete remission
Case 7	8 weeks	2 weeks	Complete remission
Case 8	52 weeks	156 weeks	Partial remission
Case 9	12 weeks	78 weeks	Complete remission
Case 10	4 weeks	5 weeks	Complete remission

Complete remission: complete resolution of pruritus and skin lesions (otherwise it is partial remission).
